# Association of vitamin K, fibre intake and progression of periodontal attachment loss in American adults

**DOI:** 10.1186/s12903-023-02929-9

**Published:** 2023-05-17

**Authors:** Yuanyuan Chuai, Bichong Dai, Xiaoyun Liu, Menglin Hu, Yuanyin Wang, Hengguo Zhang

**Affiliations:** 1 Key Laboratory of Oral Diseases Research of Anhui Province, College & Hospital of Stomatology, Anhui Medical University, Hefei, 230032 China; 2 Department of Oral and Maxillofacial Surgery, College & Hospital of Stomatology, Anhui Medical University, Hefei, 230032 China; 3 Department of Dental Implantology, College & Hospital of Stomatology, Anhui Medical University, Hefei, 230032 China

**Keywords:** Periodontitis, Attachment loss, Vitamin K, Dietary fibre, NHANES

## Abstract

**Background:**

Periodontitis-related attachment loss is accompanied by mucosal bleeding and inflammatory lesions. Dietary vitamin K and fibre intake are known to be correlation factors of haemostasis and anti-inflammation, respectively.

**Objective:**

To explore the association between severe periodontal attachment loss and vitamin K or fibre intake in American adults.

**Methods:**

A cross-sectional analysis was conducted including 2747 males and 2218 females in the National Health and Nutrition Examination Surveys (NHANES) from 2009 to 2014. The number of teeth with severe periodontal attachment loss (above 5 mm attachment loss) was used as the dependent variable. The main independent variables included the intake of vitamin K and dietary fibre. The association among variables was examined using multivariable linear regression models, hierarchical regression, fitted smoothing curves, and generalized additive models.

**Results:**

Based on the indicators of 4965 subjects, we found that severe attachment loss tended to occur in elderly individuals or males and was accompanied by less intake of vitamin K or dietary fibre, as well as lower educational qualification. Vitamin K intake was stably negatively associated with attachment loss progression in each multivariable linear regression model. In subgroup analyses, a negative association between fibre intake and attachment loss progression was identified in all races except blacks (β = 0.0005, 95% CI: -0.0005 to 0.0016). The relationship between fibre intake and attachment loss progression was a broad U-shaped curve (inflection point: 753.4 mg), which especially manifested in males (inflection point: 967.5 mg).

**Conclusion:**

There was an inverse association between vitamin K intake and the progression of periodontal attachment loss in American adults, while dietary fibre should be moderate in intake (below 753.4 mg), especially in males (below 967.5 mg).

**Supplementary Information:**

The online version contains supplementary material available at 10.1186/s12903-023-02929-9.

## Introduction

Periodontitis is a widely distributed and high-incidence oral inflammatory disease caused by complex factors [1]. Dental plaque is the initiating factor of periodontitis [2], and the severity of periodontitis depends on environmental and host risk factors, including diabetes, smoking, obesity, genetic factors, and undernutrition [3]. The initial manifestations of progressive periodontitis are bleeding gums and local inflammation [4]. As inflammation develops, chewing pressure exceeds the periodontal load, leading to alveolar bone destruction and tooth loss [5, 6]. Meanwhile, vitamin K and fibre intake are known to be correlation factors of haemostasis [7] and anti-inflammatory effects [8], respectively. Previous studies have shown that periodontitis is closely related to nutritional intake [9], dietary habits [10], and lifestyle [11]. However, whether dietary intake acts as an accurate indicator for periodontitis progression remains unclear.

Vitamin K is a fat-soluble clotting vitamin [12], and its deficiency can lead to massive bleeding [13], bone dysplasia [14], osteoporosis [15], and cardiovascular diseases [16]. Vitamin K prevents gingival bleeding through posttranslational modification of glutamate residues in coagulation factors and high binding to phospholipid membrane regions [17, 18]. In addition, fibre consumption, such as whole-grain intake, may reduce the risk of periodontitis by increasing insulin sensitivity and reducing oxidative stress and cytokine production [19]. Furthermore, fibre can be fermented by gut microbes to form short-chain fatty acids, which have anti-inflammatory properties [20]. Interestingly, dietary fibre intake is closely related to the absorption of vitamin K in humans, especially vitamin K1 [21]. To indicate the occurrence and progression of severe periodontitis, it is necessary to explore the potential relationship between vitamin K or dietary fibre intake and periodontal attachment loss progression.

In this study, we hypothesized that the intake of vitamin K or dietary fibre was associated with periodontal attachment loss progression. A cross-sectional study was conducted based on the National Health and Nutrition Examination Surveys (NHANES) dataset from 2009–2014. Multiple linear regression and hierarchical regression were used to examine the association. To evaluate the nonlinear relationship, smooth curve fitting and threshold analysis were performed. This study sought to shed light on the potential food and nutrition facts of periodontitis prevention.

## Method

### Study participants

As a cross-sectional survey of nationally representative samples from 2009 to 2014, NHANES collected information about the nutritional and health status of noninstitutionalized individuals in the United States. The study was approved by the Ethics Review Committee of the National Center for Health Statistics (NCHS), and written informed consent was obtained from each participant. Adults who were over 30 years of age, had more than 1 natural tooth (except the third molar), and did not have a health condition requiring prophylactic antibiotics before periodontal probing were eligible for a full-mouth periodontal examination [22]. After excluding participants with missing data on vitamin K or dietary fibre intake (*n* = 729), 4965 respondents who were finally included in the analysis underwent a complete NHANES oral health periodontal examination, and their nutritional status and periodontal examination data were analysed. All measurements were recorded as required by the periodontal classification algorithm, and complete data from the NHANES questionnaire were obtained.

### Periodontal examination

Periodontal attachment loss was identified by dental examiners who were dentists (D.D.S./D.M.D.) licenced in at least two U.S. states. All oral health assessments took place in a designated room at the mobile examination centre (MEC). According to the periodontitis guidelines in 2018, stage I periodontitis was defined as attachment loss (AL) ≤ 2 mm and ≥ 1 mm. Stage II was defined as AL ≤ 4 mm and ≥ 3 mm. Stage III and IV were defined as AL ≥ 5 mm. Attachment loss involving ≥ 30% of teeth was defined as generalized periodontitis attachment loss. In this study, a CPI periodontal probe was used for periodontal health assessment. Measurements were made at 6 sites around each tooth (mesio-, mid-, and distobuccal; mesio-, mid-, and distolingual) for all teeth, excluding third molars. Two measurements were made at each periodontal site, one for gingival recession, the distance from the free gingival margin to the cementoenamel junction, and the second for pocket depth (PD), the distance from the free gingival margin to the bottom of the sulcus or periodontal pocket. Clinical attachment loss (AL) was calculated as PD minus gingival recession. The number of teeth with severe periodontal attachment loss (≥ 5 mm) was used as the dependent variable [22, 23].

### Other variables

We comprehensively screened confounding factors related to periodontitis, vitamin K and dietary fibre as covariates, including age, sex, body mass index, education, and race [24–26]. In addition, the intake of vitamin K and dietary fibre were assessed by two consecutive days of dietary recalls.

### Statistical analysis

We performed all statistical analyses by using R (http://www.R-project.org), with statistical significance set at *P* < 0.05. All estimates were calculated by using sample weights following the analytical guidelines edited by the NCHS because the goal of the NHANES is to produce data representative of the civilian noninstitutionalized US population. The multivariate linear regression model and subgroup analysis were utilized to examine the relationship between vitamin K and dietary fibre intake and periodontal attachment loss. Three multiple linear regression models were constructed: Model 1, which did not adjust for covariates; Model 2, which adjusted for age, sex, and race; and Model 3, which adjusted for all covariables listed in Table 1. The nonlinear relationship was solved by smooth curve fitting and threshold analysis.

**Table 1:** Characteristics of the participants

Quartiles of PERIO	Q1 (*n* = 967)	Q2 (*n* = 1073)	Q3 (*n* = 1658)	Q4 (*n* = 1267)	*P* value
Age (years)	42.3413 ± 10.6764	54.5396 ± 14.2871	57.7376 ± 13.6435	57.8587 ± 12.3326	< 0.001
BMI	28.8750 ± 6.5105	29.9104 ± 6.4273	29.6258 ± 6.8878	29.0708 ± 6.6225	< 0.001
Vitamin K	5.9959 ± 1.1846	5.7870 ± 1.309864	5.7358 ± 1.2727	5.6458 ± 1.2683	< 0.001
Fibre	7.3291 ± 2.3188	6.8323 ± 2.4048	6.7402 ± 2.4201	6.5978 ± 2.4502	< 0.001
Gender					< 0.001
Male	416 (43.0196%)	550 (51.2582%)	925 (55.7901%)	856 (67.5612%)	
Female	551 (56.9804%)	523 (48.7418%)	733 (44.2099%)	411 (32.4388%)	
Race					< 0.001
Mexican American	101 (10.4447%)	167 (15.5638%)	260 (15.6815%)	254 (20.0474%)	
Other Hispanics	74 (7.6525%)	125 (11.6496%)	172 (10.3739%)	109 (8.6030%)	
Non-Hispanic Whites	551 (56.9804%)	431 (40.1678%)	688 (41.4958%)	452 (35.6748%)	
Non-Hispanic Blacks	117 (12.0993%)	240 (22.3672%)	397 (23.9445%)	335 (26.4404%)	
Other races	124 (12.8232%)	110 (10.2516%)	141 (8.5042%)	117 (9.2344%)	
Education					< 0.001
Below Grade 9	36 (3.7736%)	101 (9.6651%)	174 (10.6880%)	183 (14.7343%)	
Grades 9–11	47 (4.9266%)	140 (13.3971%)	278 (17.0762%)	238 (19.1626%)	
High school graduate	130 (13.6268%)	235 (22.4880%)	402 (24.6929%)	349 (28.0998%)	
College/AA degree	279 (29.2453%)	286 (27.3684%)	443 (27.2113%)	298 (23.9936%)	
College degree or above	460 (48.2180%)	281 (26.8900%)	320 (19.6560%)	170 (13.6876%)	
Refused to answer	1 (0.1048%)	0 (0.0000%)	3 (0.1843%)	0 (0.0000%)	
Do not know	1 (0.1048%)	2 (0.1914%)	8 (0.4914%)	4 (0.3221%)	

*BMI* Body mass index, *vitamin K* (log2-transformed), *fibre* (log2-transformed)

Mean ± SD for continuous variables: *P* value was calculated by the weighted linear regression model

% for categorical variables: *P* value was calculated by weighted chi-square test

## Results

The baseline characteristics of the subjects (2747 males and 2218 females) are shown in Table 1. All participants were separated into four groups according to the quartile of severe attachment loss teeth counts (Q1: 0, Q2: 1, Q3: 2 to 5, and Q4: 6 to 27). Generalized periodontal attachment loss tended to occur in elderly individuals or males and was accompanied by less intake of vitamin K and dietary fibre, as well as lower educational qualification. From Q1 to Q4, the proportion of Mexican Americans and blacks gradually increased.

The results of the multivariate regression analyses are presented in Tables 2 and 3. In Model 1, vitamin K (β = -0.2477, 95% CI: -0.3312 to -0.1643, *p* < 0.000001) and fibre intake (β = -0.2812, 95% CI: -0.3641 to -0.1984, *p* < 0.000001) were negatively correlated with attachment loss progression. After adjustment for confounders, those positive associations were stable in Model 2 (*P* < 0.000001) and Model 3 (*P* < 0.01). In the subgroup analysis stratified by sex and race, the negative association between vitamin K and attachment loss progression was stable. However, fibre intake was negatively associated with attachment loss progression in all races except blacks.

**Table 2:** Association between vitamin K intake and periodontal attachment loss progression

	Model 1 β (95% CI) *P* value	Model 2 β (95% CI) *P* value	Model 3 β (95% CI) *P* value
Vitamin K	-0.0014 (-0.0026, -0.0002) 0.022052	-0.0016 (-0.0028, -0.0005) 0.006438	-0.0009 (-0.0021, 0.0002) 0.123073
Quartiles of Vitamin K			
Q1 (0.75–31.20 mg)	0	0	0
Q2 (31.30–53.10 mg)	-0.0388 (-0.1223, 0.0446) 0.361802	-0.0686 (-0.1441, 0.0068) 0.074658	-0.0271 (-0.1010, 0.0468) 0.472478
Q3 (53.15–93.35 mg)	-0.1991 (-0.2825, -0.1157) 0.000003	-0.1894 (-0.2649, -0.1138) < 0.000001	-0.1058 (-0.1800, -0.0316) 0.005237
Q4 (93.45–1605.50 mg)	-0.2477 (-0.3312, -0.1643) < 0.000001	-0.2342 (-0.3100, -0.1584) < 0.000001	-0.1181 (-0.1930, -0.0433) 0.001982
P trend	< 0.001	< 0.001	0.010
Stratified by gender			
Male	-0.0012 (-0.0028, 0.0005) 0.173894	-0.0012 (-0.0028, 0.0004) 0.146590	-0.0005 (-0.0021, 0.0011) 0.531508
Female	-0.0023 (-0.0040, -0.0007) 0.005283	-0.0023 (-0.0040, -0.0007) 0.004573	-0.0014 (-0.0030, 0.0002) 0.084140
Stratified by race			
Mexican American	-0.0020 (-0.0075, 0.0034) 0.462763	-0.0026 (-0.0079, 0.0027) 0.338067	0.0016 (-0.0070, 0.0038) 0.557883
Other Hispanics	-0.0032 (-0.0073, 0.0009) 0.123363	-0.0022 (-0.0061, 0.0018) 0.286774	-0.0009 (-0.0048, 0.0031) 0.669704
Non-Hispanic Whites	-0.0013 (-0.0030, 0.0004) 0.143690	-0.0015 (-0.0032, 0.0002) 0.083340	-0.0002 (-0.0019, 0.0015) 0.806674
Non-Hispanic Blacks	-0.0006 (-0.0029, 0.0017) 0.592771	-0.0014 (-0.0037, 0.0008) 0.199793	-0.0017 (-0.0039, 0.0005) 0.131520
Other races	-0.0017 (-0.0053, 0.0020) 0.369406	-0.0016 (-0.0052, 0.0020) 0.386952	-0.0003 (-0.0039, 0.0032) 0.851076

Model 1: No covariates were adjusted

Model 2: Age, gender, and race were adjusted

Model 3: Adjusted for all variables listed in Table 1

**Table 3:** Relationship between dietary fibre intake and periodontal attachment loss progression

	Model 1 β (95% CI) *P* value	Model 2 β (95% CI) *P* value	Model 3 β (95% CI) *P* value
Fibre	-0.0009 (-0.0014, -0.0005) 0.000008	-0.0008 (-0.0012, -0.0004) 0.000050	-0.0008 (-0.0012, -0.0004) 0.000190
Quartiles of fibre			
Q1 (0.00–19.35 mg)	0	0	0
Q2 (19.40–238.65 mg)	-0.0288 (-0.1123, 0.0546) 0.498731	-0.0448 (-0.1204, 0.0308) 0.245495	-0.0080 (-0.0815, 0.0656) 0.832138
Q3 (238.70–469.55 mg)	-0.1536 (-0.2374, -0.0697) 0.000333	-0.1545 (-0.2308, -0.0781) 0.000075	-0.1249 (-0.1996, -0.0502) 0.001051
Q4 (470.25–2428.85 mg)	-0.2812 (-0.3641, -0.1984) < 0.000001	-0.2170 (-0.2924, -0.1415) < 0.000001	-0.1695 (-0.2429, -0.0960) 0.000006
P trend	< 0.001	< 0.001	< 0.001
Stratified by gender			
Male	-0.0012 (-0.0018, -0.0007) 0.000009	-0.0010 (-0.0015, -0.0005) 0.000298	-0.0010 (-0.0015, -0.0004) 0.000374
Female	-0.0008 (-0.0014, -0.0002) 0.008639	-0.0005 (-0.0011, 0.0001) 0.116644	-0.0004 (-0.0010, 0.0002) 0.232311
Stratified by race			
Mexican American	-0.0012 (-0.0023, -0.0001) 0.029996	-0.0012 (-0.0023, -0.0002) 0.020879	-0.0012 (-0.0023, -0.0002) 0.023186
Other Hispanics	-0.0009 (-0.0020, 0.0003) 0.134178	-0.0008 (-0.0019, 0.0002) 0.128930	-0.0008 (-0.0018, -0.0003) 0.159892
Non-Hispanic Whites	-0.0013 (-0.0019, -0.0008) 0.000002	-0.0012 (-0.0017, -0.0007) 0.000014	-0.0012 (-0.0018, -0.0007) 0.000010
Non-Hispanic Blacks	0.0004 (-0.0007, 0.0014) 0.480656	0.0003 (-0.0007, 0.0013) 0.543631	0.0005 (-0.0005, 0.0016) 0.293950
Other races	-0.0006 (-0.0022, 0.0011) 0.503325	-0.0005 (-0.0021, 0.0011) 0.568293	-0.0003 (-0.0019, 0.0013) 0.714169

Model 1: No covariates were adjusted

Model 2: Age, gender, and race were adjusted

Model 3: Adjusted for all variables listed in Table 1

**Fig. 1:**
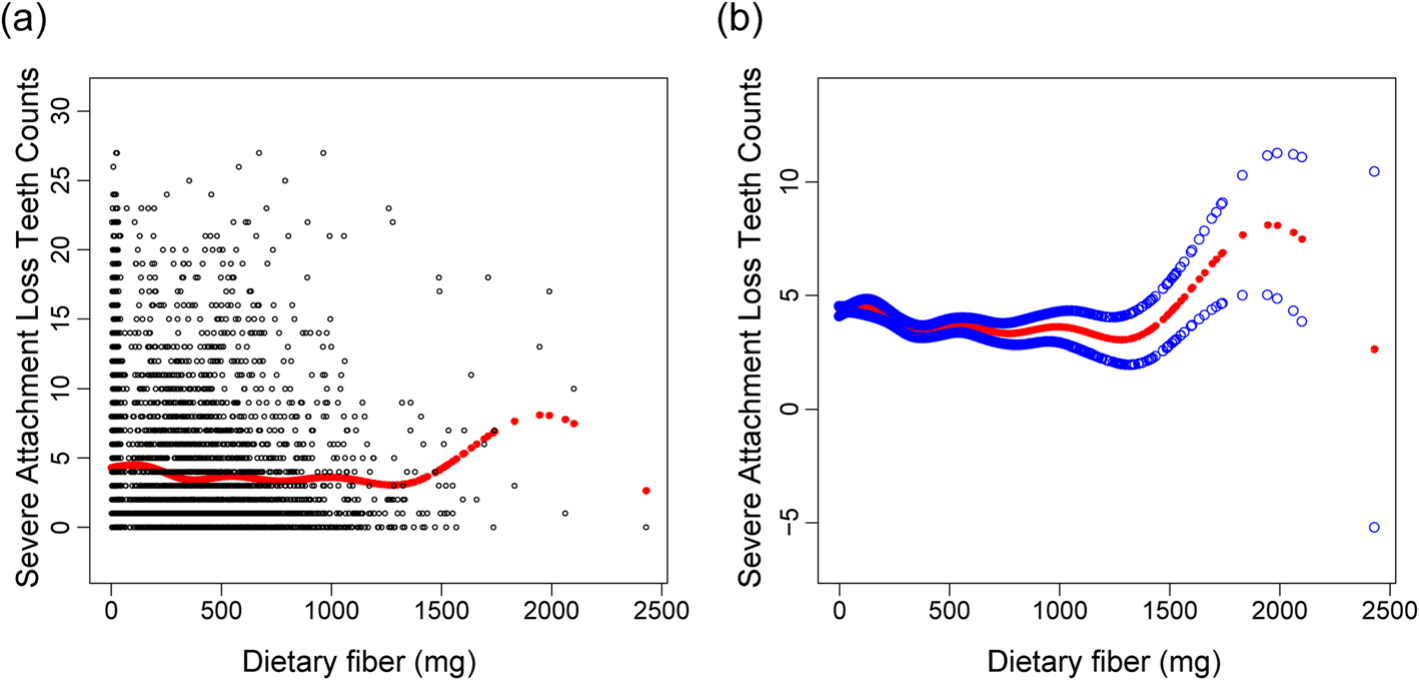
Association between dietary fibre intake and loss of periodontal attachment. **a** Each black dot represents a sample. **b** The solid line represents the smooth curve fitting between variables. The blue bands represent the 95% confidence intervals of the fit

**Fig. 2:**
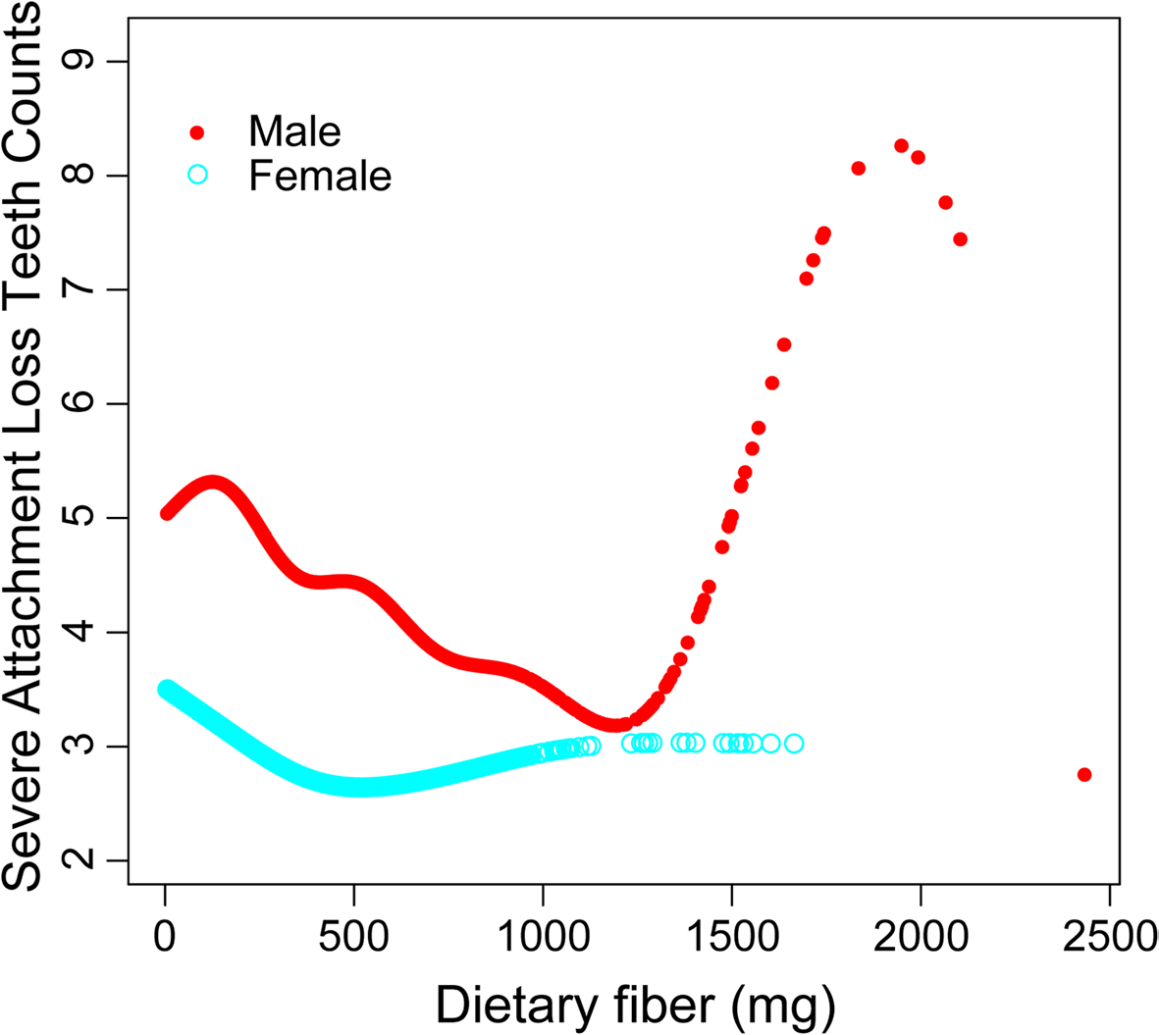
Association between dietary fibre intake and loss of periodontal attachment, stratified by sex. Adjusted for all variables listed in Table 1

In addition, the weighted generalized additive models and smooth curve fitting showed the nonlinear relationship between dietary fibre intake and attachment loss progression. More details of vitamin K and attachment loss progression are shown in Figures S1 and S2. By two-piecewise linear regression models, the inflection point of dietary fibre intake was 753.4 mg (Table 4). For dietary fibre intake < 753.4 mg, every 1 mg increase in dietary fibre intake was correlated with 0.0016 (95% CI:—0.0021 to—0.0010) fewer severe attachment loss teeth. By comparison, for individuals with dietary fibre intake > 753.4 mg, every 1 mg increase in vitamin K or dietary fibre intake was significantly associated with 0.0015 (95% CI: 0.0001 to 0.0029) more severe attachment loss teeth. In addition, the relationship between fibre intake and attachment loss progression showed a broad U-shaped curve (Fig. 1), especially for males (inflection point: 967.5 mg) (Fig. 2, Table 5).

**Table 4:** Two-piecewise regression analysis of the effect of dietary fibre intake on the progression of attachment loss

Attachment loss progression	Adjusted β (95% CI), *P* value
Fitting by the standard linear model	-0.0009 (-0.0014, -0.0005) < 0.0001
Fitting by the two-piecewise linear model	
Inflection point	753.4 mg
Dietary fibre < 753.4 (mg)	-0.0016 (-0.0021, -0.0010) < 0.0001
Dietary fibre > 753.4 (mg)	0.0015 (0.0001, 0.0029) 0.0362
Log likelihood ratio	< 0.001

**Table 5:** Two-piecewise regression analysis of the effect of dietary fibre intake on the progression of attachment loss in males and females

Attachment loss progression	Males β (95% CI), *P* value	Females β (95% CI), *P* value
Fitting by the standard linear model	-0.0012 (-0.0018, -0.0007) < 0.0001	-0.0008 (-0.0014, -0.0002) 0.0086
Fitting by the two-piecewise linear model		
Inflection point	967.5 mg	18.05 mg
Dietary fibre < Inflection point	-0.0018 (-0.0024, -0.0011) < 0.0001	-0.1180 (-0.1743, -0.0618) < 0.0001
Dietary fibre > Inflection point	0.0022 (-0.0003, 0.0047) 0.0863	-0.0002 (-0.0008, 0.0005) 0.6687
Log likelihood ratio	0.006	< 0.001

## Discussion

As the sixth most prevalent disease in the world, periodontitis causes alveolar bone resorption and tooth loss in adults [27]. Severe alveolar bone loss adds to the challenge of implant restoration and financial burden [28]. Therefore, exploring the risk factors for periodontitis is crucial to alveolar bone health and tooth survival [29]. Previous studies focused on the mechanism and treatment of severe periodontitis, including genetics [30], protein expression [31], and immune response [32]. For example, IL-23-dependent IL-17 is a powerful proinflammatory mediator that leads to bacterial overgrowth that causes periodontal disease [33]. Another study found that the modified MCPIP-1 and MALT-1 response and protein expression induced by periodontitis-related bacteria may be part of the pathogenesis of periodontitis [34]. A recent study found that circRNA forms a circRNA–miRNA‒mRNA network during the osteogenic differentiation of periodontal stem cells, which has the prospect of synchronous periodontal diagnosis and regeneration [30]. Multivariate logistic regression analyses indicated that abundant vitamin K and moderate dietary fibre are beneficial to periodontal health.

In addition to the haemostatic effects [13], vitamin K can also prevent bone health by taking in carboxylated vitamin K-dependent proteins, such as osteocalcin and stromal glass protein [15]. The lack of a balanced diet rich in vitamins and minerals predisposes individuals to inflammatory diseases, such as obesity, diabetes, and periodontitis [35]. It has been suggested that vitamin K2, a cofactor of vitamin D and calcium, can support bone health by promoting osteoblast differentiation and upregulating transcription [36]. It can also regulate immune cells that cause inflammation by reducing the production of inflammatory markers and therefore reducing inflammation [37]. A previous study showed that adequate vitamin K administration reduces osteoclast and alveolar bone resorption in patients with experimental periodontitis [38]. Multivariable linear regression results also suggested that vitamin K intake was stably negatively associated with attachment loss progression.

Healthy eating habits, especially increased dietary fibre intake, may improve periodontitis markers by reducing serum CRP levels [39]. Recent research identified that higher levels of fibre intake were inversely associated with periodontitis [40]. However, the study showed that dietary fibre intake was negatively correlated with periodontal attachment loss, and the threshold reverse effect occurred when a certain value was reached (inflection point = 753.4 mg). Due to the degeneration of periodontal membrane sensory function in patients with periodontitis, excessive intake of dietary fibre leads to pressure concentration, tooth abrasion and alveolar bone resorption [41]. The inflection point of fibre intake in the females was 18.05 mg, and the inflection point for the males was 967.5 mg. Due to the more powerful chewing function of males, an increased risk of attachment loss occurs with excessive fibre intake [42].

Although the current study demonstrated the association of vitamin K, dietary fibre intake and the progression of periodontal attachment loss, there are also several limitations. First, as a cross-sectional study, it was impossible to ascertain temporality between exposure and outcomes. Second, as we focused on the potential food and nutrition facts of periodontitis prevention, the intake of vitamin K was not equal to circulatory vitamin K, and we will further explore the association of circulatory vitamin K and periodontal attachment loss. Moreover, due to the occlusion function of participants being unknown, the relevant potential factors affecting mastication should be distinguished in future studies. Third, vitamin K and dietary fibre intake in this study was determined by a two-day dietary review interview with participants, which may be subject to recall bias. Fourth, the types of dietary fibre are diverse and need to be further divided in the future.

## Conclusion

Vitamin K intake was inversely associated with the progression of periodontal attachment loss among American adults. Meanwhile, a broad U-shaped relationship (inflection point, 753.4 mg) between fibre intake and attachment loss progression was identified, particularly in males (inflection point, 967.5 mg). The study guides the potential role of dietary habits in community oral health prevention.

## Supplementary Information

**Additional file 1: Figure S1.** Association between Vitamin K intake and loss of periodontal attachment. Each black dot represents a sample. The solid line represents the smooth curve fitting between variables. The blue bands represent the 95% confidence intervals of the fit.

**Additional file 2: Figure S2. **Association between vitamin K and loss of periodontal attachment, stratified by sex. Adjusted for all variate lists in Table 1.

## Data Availability

Publicly available datasets were analysed in this study. Data used for this study are available on the NHANES website: https://wwwn.cdc.gov/nchs/nhanes/.
