# Supplementary figures and images for: Association of vitamin K, fibre intake and progression of periodontal attachment loss in American adults
Source: BMC Oral Health. 2023 May 17;23:303. doi: 10.1186/s12903-023-02929-9 (PMC10193670; doi:10.1186/s12903-023-02929-9)

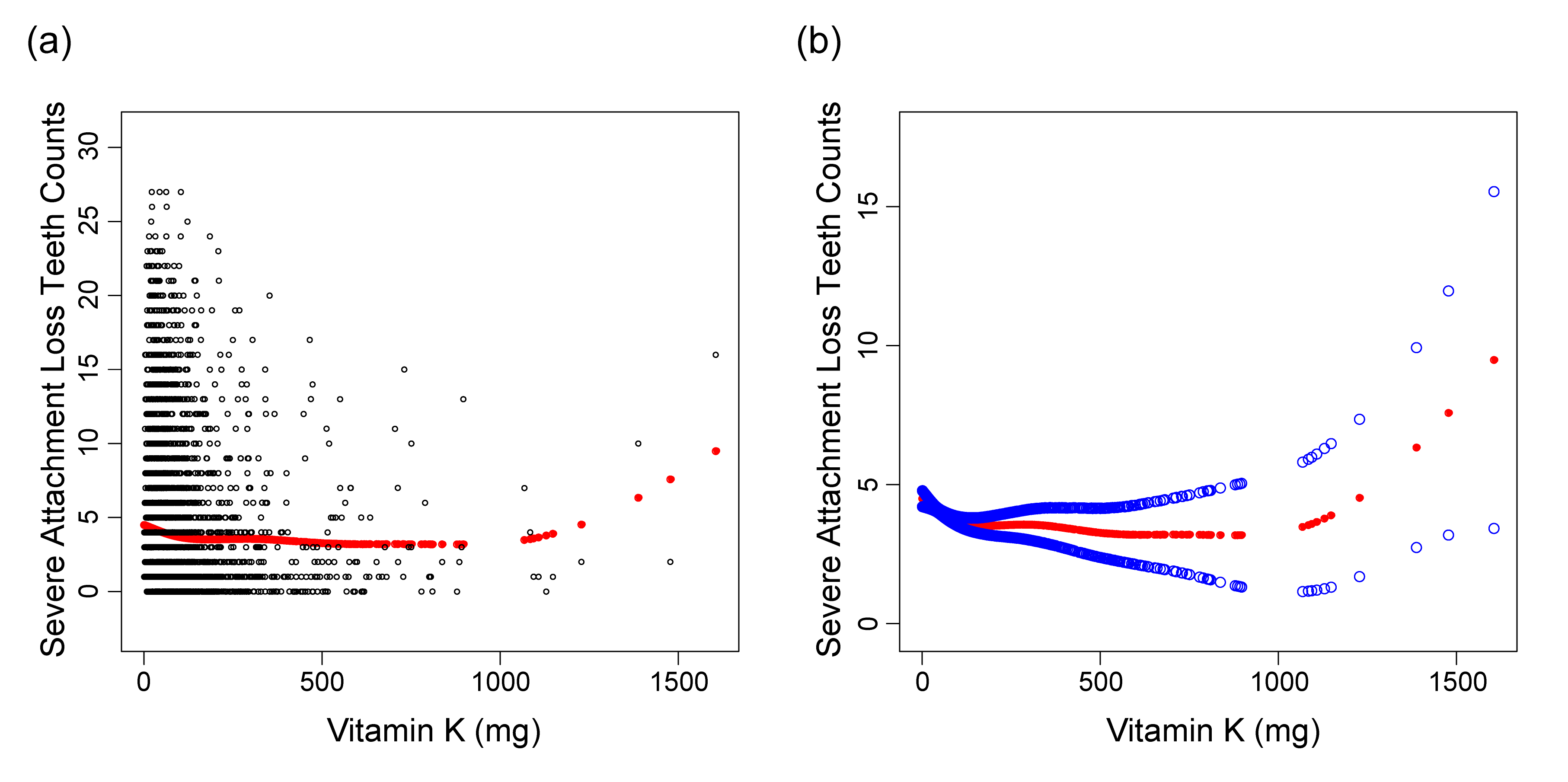

Supplement: Supplementary file 1 — Additional file 1: Figure S1. Association between Vitamin K intake and loss of periodontal attachment. Each black dot represents a sample. The solid line represents the smooth curve fitting between variables. The blue bands represent the 95% confidence intervals of the fit. [file 12903_2023_2929_MOESM1_ESM.tif]

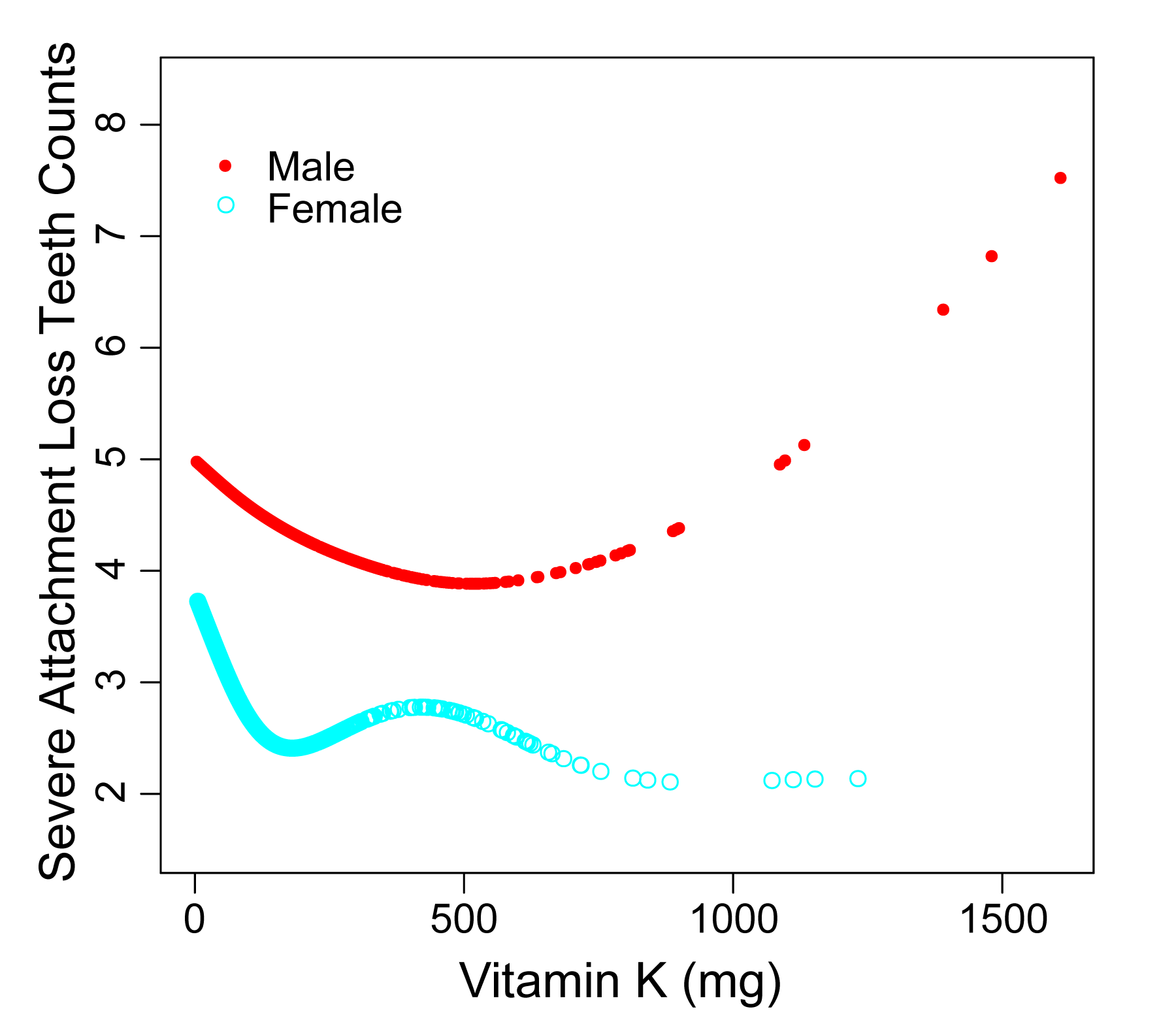

Supplement: Supplementary file 2 — Additional file 2: Figure S2. Association between vitamin K and loss of periodontal attachment, stratified by sex. Adjusted for all variate lists in Table 1. [file 12903_2023_2929_MOESM2_ESM.tif]
